# Pre-infection administration of asiatic acid retards parasitaemia induction in *Plasmodium berghei* murine malaria infected Sprague-Dawley rats

**DOI:** 10.1186/s12936-016-1278-6

**Published:** 2016-04-21

**Authors:** Greanious Alfred Mavondo, Blessing Nkazimulo Mkhwananzi, Musa Vuyisile Mabandla

**Affiliations:** Discipline of Human Physiology, School of Laboratory Medicine and Medical Sciences, College of Health Sciences, University of KwaZulu Natal, Westville Campus, Durban, 4000 South Africa

**Keywords:** Asiatic acid, Malaria parasitaemia, *Plasmodium berghei*, Prophylaxis treatment

## Abstract

**Background:**

Malaria prevention has remained a critical area in the absence of efficacious vaccines against malaria. Drugs currently used as chemotherapeutics are also used in chemoprophylaxis increasing possible drug resistance. Asiatic acid is a natural phytochemical with oxidant, antioxidant and anti-inflammatory properties with emerging anti-malarial potential. The influence of asiatic acid administration prior to *Plasmodium berghei* infection of Sprague-Dawley rats on parasitaemia induction is here reported.

**Methods:**

Sprague-Dawley rats (90–120 g) were administered with asiatic acid (10 mg/kg) 48 h before intraperitoneal infection with *P. berghei*. Parasitaemia induction and progression, food and water intake as well as weight were compared to 30 mg/kg chloroquine-treated and infected control rats during sub-chronic studies (21 days).

**Results:**

Asiatic acid pre-infection administration preserved food and water intake as well as increase in percentage weight gain of infected animals. In pre-infection treated animals, the pre-patent period was extended to day 6 from 72 h. Asiatic acid suppressed parasitaemia while oral chloroquine (30 mg/kg) did not influence malaria induction.

**Conclusions:**

Per-oral, pre-infection, asiatic acid administration influenced parasitaemia patency and parasitaemia progression, food, water, and weight gain percentage. This may suggest possible chemoprophylaxis effects of asiatic acid in malaria.

## Background

Chemoprophylaxis can either be causal prophylaxis (absolutely prevents patent malaria development by eradicating liver stage parasites) or parasitaemia and malaria symptom suppression, referred to as suppressive or clinical prophylaxis, where blood stage parasites are destroyed from circulation [1]. Drugs used as prophylaxis need to be long acting or have longer half-lives as frequent dosing may lead to non-compliance [2, 3]. The drug also needs to be palatable and tolerable, facets absent in most prophylaxis drugs with phytochemical origins [4, 5]. In Uganda, an infusion of *Artemisia annua* consumed once weekly reduced risk of *Plasmodium falciparum* infection episodes due to as yet an unidentified constituent [6] with a longer half-life than artemisinin. However, its only drawback is the bitterness [7]. Fascinating results have also started to emerge where triterpenes oleanolic acid (OA) [8] and maslinic acid (MA) [9] have shown amelioration of metabolic dysfunction in malaria. These triterpenes have been reported to have anti-inflammatory activities as well [10, 11]. These findings suggest that other triterpenes may also have anti-malarial activity giving rise to this current investigation of asiatic acid (AA) as a potential anti-malarial. Asiatic acid is an intriguing molecule with antioxidant and pro-oxidant [12], anti-inflammatory [13] and immunomodulatory [14] activities. Chemically known as (4α)-2α, 3β, 23-trihydroxy-urs-12-en-28-oic acid, having redox reaction capability, amphiphilic with a hydrogen bond donor/acceptor ratio of 7.1/4.172 [15], AA has potential for anti-disease properties in malaria. Indeed, ongoing (unpublished) anti-malarial work with the triterpene AA has shown that the phytochemical has an abrupt or sudden parasite killing effect during the post-dosing period in infected Sprague-Dawley (SD) male rats. In those experiments high percentage parasitaemia seemed to just ‘disappear’ from subsequent peripheral slides without a predictable gradual decline seen with other anti-malarial drugs. With this in mind, the aim of the study was to establish whether the amphiphilic triterpenoid could have cumulative long-acting pharmacodynamics potentially useful for malaria chemoprevention. Findings on the influence of pre-infection administration of AA on the retardation of malaria development in *Plasmodium berghei*-infected SD male rats by monitoring malaria infection, percentage parasitaemia, as well as food and water intake are here reported.

## Methods

### Drugs and chemicals

The initial AA (500 mg) of 97 % purity used in the preliminary studies was a kind donation from Prof Van Heerden (University of KwaZulu Natal). Further quantities of AA (97 % purity) were purchased together with Giemsa stain, dimethyl sulfoxide (DMSO), chloroquine diphosphate (CHQ) from Sigma-Aldrich (St. Louis, MI, USA). All other chemicals and reagents were of analytical grade.

### Animals

Male SD rats weighing 90–120 g were obtained from the Biomedical Research Animal Unit (BRU) of the University of KwaZulu where they were bred and housed for the entire experiment period. The animals were kept under maintained laboratory conditions of constant temperature (22 ± 1 °C); Co_2_ (<5000 ppm), humidity of 55 ± 5 % and illumination (12 h light/dark cycles). Food, standard rat chow (Meadows Feeds, Pietermaritzburg, South Africa) and water were supplied ad libitum. All animals were sacrificed by day 21 through exposure to halothane for 3 min via an anaesthetic gas chamber (100 mg/kg). All experiments and protocols were reviewed and approved by the animal ethics committee of the University of KwaZulu Natal (UKZN) with ethical clearance numbers 079/14/Animal and 013/15/Animal issued.

### Murine malaria model

Chloroquine-susceptible strain of *P. berghei* ANKA, was a kind donation from Prof Peter Smith (University of Cape Town, Division of Clinical Pharmacology, South Africa). The parasite was sub-cultured in SD rats and harvested into Na_2_EDTA whole blood. The blood was washed and stored in freeze media containing 30 % glycerol at −80 °C until used.

### Experimental design

Animal groups (n = 6) were divided according to whether they were infected or received treatment. Animals treated with CHQ (30 mg/kg) served as the positive control. The groups were as follows:

Non-infected treated control (NIC)

Infected non-treated control (IC)

Infection groups treated with CHQ 30 mg/kg (30CHQ)

Infected groups treated with AA 10 mg/kg (10AA).

### Monitoring of physicochemical properties

Six animals per group were housed individually in Makrolon polycarbonate metabolic cages (Techniplast, Labotec, South Africa) with food and water availed to them ad libitum. Food, water intake and weight gain were determined gravimetrically every other day at 09.00 h.

### Pre-infection oral administration

AA (10 mg/kg) and CHQ (30 mg/kg) were administered on successive days (days 0–5). AA was administered once daily (09.00) according to the posology developed for triterpenes [11, 16–18] and what doses others have advocated for treatment of other conditions [12, 19, 20]. CHQ was administered twice daily (09.00 and 16.00) for the same duration as AA. CHQ dose is a standard regimen for malaria prophylaxis in combination with doxycycline or proguanil [1]. A ball-tipped, 18-gauge gavage needle (Kyron Laboratories (Pty) Ltd, Benrose, South Africa) attached to a 1-ml syringe was used intragastric (ig) to deliver AA and CHQ.

### Induction of parasitaemia

*Plasmodium berghei* (10^5^ parasitized red blood cells (pRBCs) suspension in saline) was inoculated intraperitoneal (ip) [21]. Control animals received equivalent amounts of saline. Animals were inoculated 48 h after AA or CHQ administration. Administration of AA and CHQ was continued up to day 5 giving a total of 5 days administration inclusive of the pre-infection period.

### Evaluation of parasitaemia

Appearance of parasites in blood after ip inoculation takes 2 to 3 days [22]. Pre-patent period was expected at 72 h post-infection and a stable parasitaemia at 15–20 % on day 7 [23]. After inoculation, parasitaemia was monitored at 72 h (pre-patent period) and every third day during the patent period [24] thereafter, until day 21. A 15–20 % parasitaemia was considered as stable state severe malaria (SM) capable of inducing severe malaria anaemia (SMA). Stable state malaria was expected at day 7.

### Influence of AA on percentage parasitaemia

Giemsa staining: peripheral blood obtained through a tail prick was made into thin blood smears and stained with Giemsa stain for monitoring of percentage parasitaemia by examination under a light microscope (Olympus Cooperation, Tokyo, Japan). The actual number of pRBCs relative to 2 × 10^4^ RBCs was used to calculate parasitaemia [22].

Full blood count: To further explore the influence of AA on malaria and its co-morbidities of inflammation and SMA, white cell count (WBC) and haemoglobin estimations were made from blood obtained through cardiac puncture at days 0, 3, 9, 12, and 21 after administration of lethal anaesthesia with halothane. All IC animals were sacrificed by day 12 on ethical grounds to reduce pain and suffering using a humane method of halothane anaesthetic inhalation in gas chamber (100 mg/kg) and blood collected by cardiac puncture.

### Statistical analysis

Unless otherwise stated, data were presented as mean plus standard error of the mean (M ± SEM). Statistical comparisons was performed by one-way analysis of variance (ANOVA), followed by Tukey–Kramer multiple comparison post hoc test using Graph-pad Prism Software (version 5, GraphPad Software, San Diego, CA USA). P < 0.05 was considered statistically significant.

## Results

### Influence of AA on physicochemical properties

Table 1 shows the influence of AA administration on food and water as well as percentage weight gain. IC animals had significantly decreased water and food intake as well as body weight at day 12 compared to animals administered with AA (10 mg/kg) (*p < 0.05). CHQ treatment decreased food and water intake together with percentage weight gain when compared to AA (10 mg/kg) administration at relevant time points (**p < 0.05). Animals treated with 30CHQ had lower food and water intake as well as percentage weight gain when compared to NIC (γp < 0.05).

**Table 1:** Influence of asiatic acid (10 mg/kg) on biophysical properties compared to controls

Parameter	Protocol name		Pre-patent (D 3)	Patent/ Treatment (D7–12)	Post-treatment (D 21)
		Animal Groups			
Food intake (g/100 g)	Pre-infection per oral AA administration	NIC	10 ± 3	11 ± 2	12 ± 1
		IC	9 ± 1	6 ± 2	N/A
		30 CHQ	10 ± 1	6 ± 1	8 ± 4
		AA (10 mg/kg)	10 ± 3	9 ± 2*^,^ **	12 ± 1**
Water intake (mL/100 g/day)	Pre-infection per oral AA administration	NIC	15 ± 3	13 ± 1	16 ± 2
		IC	15 ± 2	7 ± 2	N/A
		CHQ	14 ± 1	10 ± 1	12 ± 2γ
		AA (10 mg/kg)	15 ± 3	14 ± 1*^,^ **	15 ± 2**
% body weight change	Pre-infection per oral AA administration	NIC	8 ± 3	10 ± 2	15 ± 1
		IC	8 ± 2	−4 ± 2	N/A
		CHQ	8 ± 1	5 ± 1γ	6 ± 1γ
		AA (10 mg/kg)	8 ± 4	11 ± 2*^,^ **	14 ± 1**

Changes on percentage body weight gain, food and water intake of *P. berghei*- infected treated and non-treated animals were monitored. Values are presented as mean ± SEM, (n = 6 per group)

*NIC* non infected treated control, *IC* infected non-treated control, 30*CHQ* chloroquine 30 mg/kg.

*^,^ ** p < 0.05 by comparison to the IC, CHQ, respectively

### Validation of parasitaemia

Table 2 shows the influence of AA (10 mg/kg) on pre-patent period, percentage parasitaemia. AA (10 mg/kg) administration significantly influenced prolongation of the pre-patent period, parasitaemia inhibition by day 3 while reducing percentage parasitaemia at day 7 in comparison to the IC (*p < 0.05). AA (10 mg/kg), in comparison to 30CHQ (**p < 0.05) influenced prolongation of pre-patent period, parasitaemia inhibition at day 3 and reduction of percentage parasitaemia by day 7. In comparison to the IC, the positive control 30CHQ reduced percentage parasitaemia at day 3 (*p < 0.05). Animals administered AA (10 mg/kg) did not reach stable state malaria by day 7 in comparison to IC and 30CHQ (*^,^ **p < 0.05, respectively). AA (10 mg/kg) had lower peak percentage parasitaemia compared to both the IC and 30CHQ controls (*^,^ **p < 0.05, respectively). AA (10 mg/kg) peak period (day) was statistical different compared to that of 30CHQ (**p < 0.05).

**Table 2:** Percentage parasitaemia during different time points per different groups

Protocol	Groups	Pre-patent parasitaemia (days)	Parasitaemia on day 3 (%)	Parasitaemia on day 7 (%)	Parasitaemia at peak (%)	Peak period (day)
Pre-infection AA administration	IC	2–3	5.27 ± 1.17	15.72 ± 2.98	56.52 ± 3.20	12
	30CHQ	2–3	1.167 ± 0.31	16.08 ± 1.33	22.37 ± 4.36	9
	AA10 mg/kg	6*^,^ **	0.00 ± 0.0*^,^ **	0.13 ± 2.03*^,^ **	7.51 ± ^,^ **	12**

Values are presented as mean ± SEM, (n = 6 per group)

*IC* infected non-treated control, 30*CHQ* chloroquine 30 mg/kg

*^,^ ** p < 0.05 compared to IC, 30CHQ, respectively

### Validation of asiatic acid influence on cellular morphology

**Fig. 1:**
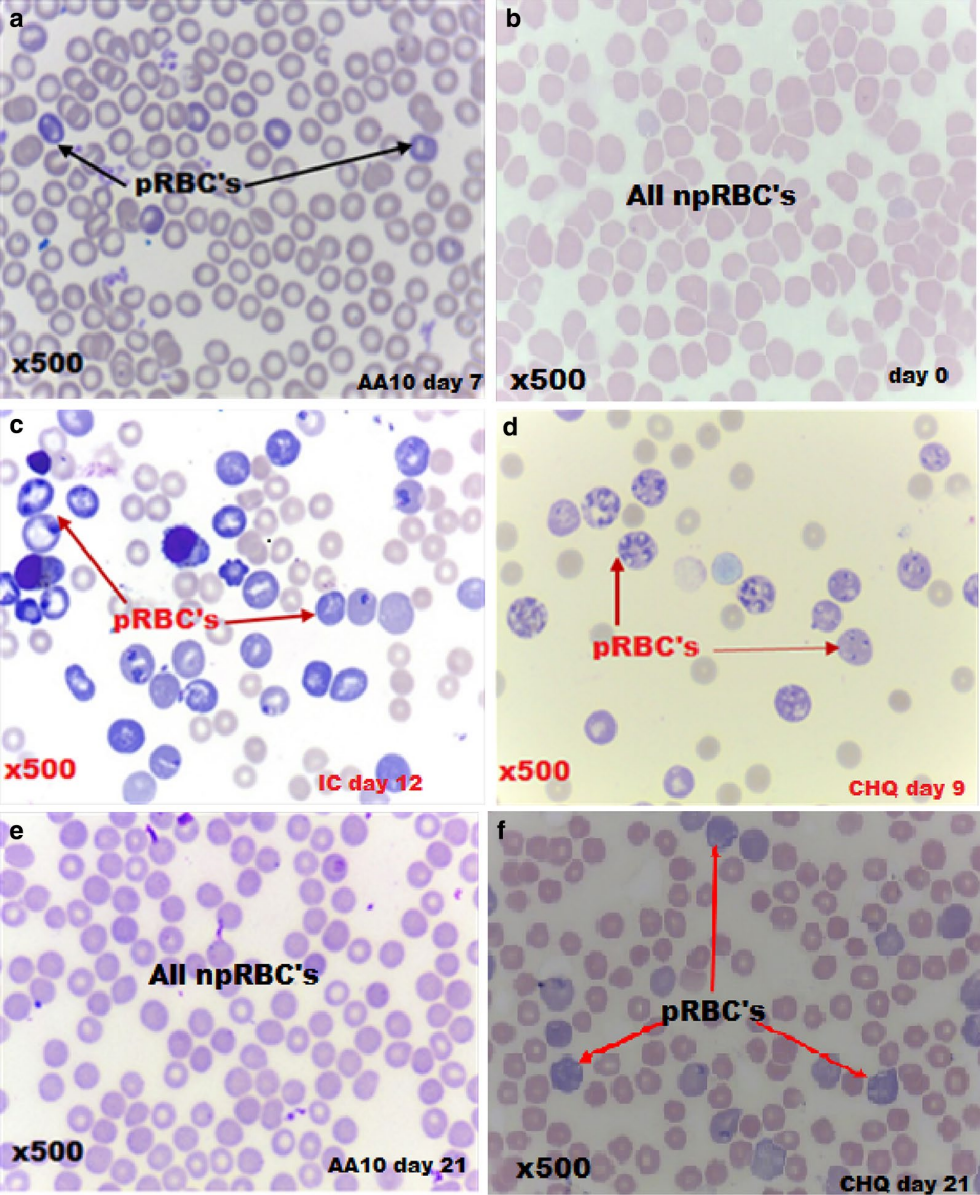
Giemsa staining showing asiatic acid (10 mg/kg) influence on cellular morphology. Micrographs were before (day 0), during (day 7) and after malarial infection (day 21) and were compared to IC (day 12) and CHQ (days 9 and 21) controls. Slide are from: **a** AA 10 adminisitered animals on day 7; **b** day 0; **c** IC day 12; **d** CHQ day 9; **e** AA 10 day 21; **f** CHQ day 21. Parasitized red blood cells (pRBCs) are indicated by *black arrows* in slide **a**, *red arrows* in slides **c**, **d** and **f**. Non-parasitized red blood cells (npRBCs) are shown in slides **b** and **e**. ×500 magnification used in all slides

As seen on Fig. 1, patent parasitaemia showed differential staining with Giemsa stain (×100 objective) where pRBCs showed as purple cells with or without parasites in them. By day 12 npRBCs in IC and CHQ groups were pail pick and reduced in number showing anaemia. There was minimum anisocytosis with slight polychromasia. Slides [C] and [D] from IC and CHQ-treated groups at days 12 and 9, respectively showed increased parasitaemia. Most cells were parasitized with visible parasite ring forms chromatin evident. Slides [E] was from AA (10 mg/kg)-administered group showing parasitaemia suppression at day 21. No pRBCs could be demonstrated in all AA-administered animals by day 21. Micrograph [E] from 30CHQ-treated animals showed that parasitaemia was still evident although significantly reduced compared to day 9.

### Asiatic acid influence on percentage parasitaemia

#### AA administration and percentage parasitaemia

**Fig. 2:**
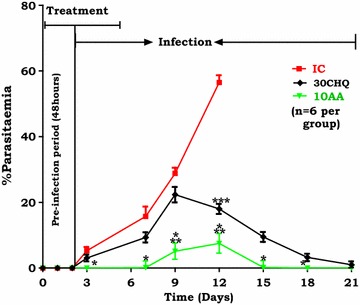
Asiatic acid (10 mg/kg) influence on percentage parasitaemia compared to controls. Values are presented as mean ± SEM, (n = 6 per group). *^,^ **p < 0.05 compared to IC and CHQ, respectively. ***p < 0.05 30CHQ compared to IC

Figure 2 shows changes in percentage parasitaemia over time. AA (10 mg/kg) administration displayed significantly lower percentage parasitaemia compared to the IC (*p < 0.05) on days 3–12. Compared to 30CHQ, AA (10 mg/kg) had lower percentage parasitaemia (***p < 0.05) throughout the 21 days of the sub-chronic study. 30CHQ treatment lowered percentage parasitaemia significantly at day 12 in comparison to the IC (***p < 0.05).

#### AA administration and percentage parasitaemia-time area under the curve

**Fig. 3:**
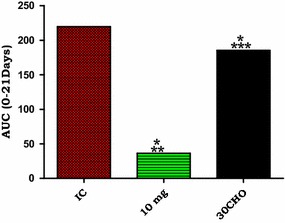
Percentage parasitaemia-time AUC _(0–21days)_ for AA 10 mg/kg compared to controls. Controls included IC AUC_0–12 days_ and CHQ AUC_0–21 days_. *AA* asiatic acid, *IC* infected non-treated control, 30*CHQ* chloroquine 30 mg/kg. Values are presented as mean ± SEM, (n = 6 per group). *^,^ **p < 0.05 compared to the IC and CHQ controls, respectively. ***p < 0.05 CHQ compared to IC

Figure 3 shows the influence of AA (10 mg/kg) on percentage parasitaemia-time area under the curve (AUC_0–21days_). AA (10 mg/kg) decreased the percentage parasitaemia-time curve significantly compared to the IC AUC_0–12days_ (*p < 0.05). Compared to 30CHQ treatment, AA (10 mg/kg) administration reduced the AUC_0–21days_ significantly (**p < 0.05) at the same time points. Compared to IC AUC_0–12days_, 30CHQ treatment reduced AUC_0–21days_ significantly (***p < 0.05). Overall, the percentage parasitaemia-time IC AUC_0–12 days_ was significantly higher than either AA (10 mg/kg) or 30CHQ AUC_0–21 days_ (*^,^ ***p < 0.05, respectively), regardless of the shorter time period.

#### AA influence on inflammation

**Fig. 4:**
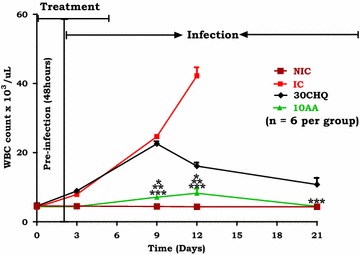
Comparison of WBC count over time in AA10 administered animals with controls. Values are presented as mean ± SEM, (n = 6 per group). *NIC* non infected treated control, *IC* infected non-treated control, 30*CHQ* chloroquine 30 mg/kg. Values are presented as mean ± SEM, (n = 6 in each group). *^,^ **^,^ *** p < 0.05 by comparison with NIC, IC and 30CHQ-treated groups

Figure 4 shows the effect of AA (10 mg/kg) on WBC count over time. AA (10 mg/kg) administration lowered WBC count significantly compared to IC (*p < 0.05). Compared to 30CHQ, AA (10 mg/kg) decreased WBC significantly (**p < 0.05). At peak percentage parasitaemia AA (10 mg/kg) administered animal had a significantly higher WBC count compared to the NIC (α p < 0.05). Treatment with 30CHQ had higher WBC counts compared to the NIC (***p < 0.05) throughout the 21-day period.

#### Influence of AA on severe malaria anaemia

**Fig. 5:**
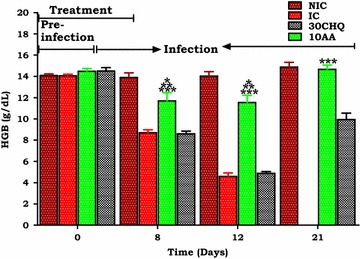
Comparison of AA (10 mg/kg) administration on haemoglobin concentration with controls. Hb was estimated in *P. berghei*- infected AA10 treated SD rats with the NIC, IC and 30CHQ controls. *NIC* non infected treated control, *IC* infected non-treated control, 30*CHQ* chloroquine 30 mg/kg. Values are presented as mean ± SEM, (n = 6 in each group). *^,^ **^,^ *** p < 0.05 compared NIC, IC and CHQ, respectively

Figure 5 shows changes in haemoglobin (Hb) with administration of AA (10 mg/kg) over time. Administration of AA (10 mg/kg) had significantly higher Hb levels compared to the IC (**p < 0.05). Compared to 30CHQ, AA (10 mg/kg) had significantly higher Hb levels (***p < 0.05) throughout the 21-day study.

## Discussion

Anecdotal information ascribes anti-malarial activity to *Centella asiatica* (CA) [25, 26] but there are no reports of chemoprophylaxis or chemotherapeutic effects of AA on malaria. There is a close resemblance in malaria pathophysiology between *P. berghei* and *P. falciparum*, the virulent species of human malaria parasites, warranting the former to be used as a safer analogue in experimental malaria. Studies have indicated, also proven by earlier research, that *P. berghei* causes SM proceeding to cerebral malaria (CM) in younger animals [23, 27–30]. Here, demonstrated is the novel influence of pre-infection po administration of AA on malaria in young, six weeks old SD rats (90–120 g) displaying hyperparasitaemia, SM and SMA.

Both murine malaria parasite (*P. berghei*) and SD rat malaria animal models conformed to a high predictive validity at days 3 and 7 in the IC and 30CHQ controls but not in the AA-administered group. Infection induction by an ip inoculation of 10^5^ of pRBCs saline suspension invariably results in SM within 2 weeks, however this did not seem to happen in animals administered with AA (10 mg/kg) during the pre-infection period of 48 h. The pre-patent duration (the time it takes for parasites to be detected in peripheral blood from the day of inoculation) is usually 72 h. This period was, however, prolonged by a further 3 days to a period beyond day 6 when AA (10 mg/kg) was administered 48 h before the animals were infected. The prevention of malaria could only be ascribed to the influence of AA as animal groups treated with CHQ (30 mg/kg) showed a malaria progression pattern as predicted [22]. Furthermore, subdued patent percentage parasitaemia, which failed to reach stable state malaria in AA (10 mg/kg) (Table 2)-administered groups, shows a possible cumulative concentration or effect of AA which continued to interact with the parasite well after administration of AA had ceased. This phenomenon was supported by observations of the percentage parasitaemia-time curve in this study (Fig. 2) which clearly showed AA10 administration causing a diminutive infection-time course compared to that of the IC and the CHQ controls. Notably, the IC percentage parasitaemia-time area under the curve covered only a maximum of 12 days, yet it was several times higher than that of AA10. Although others factors may be at play, the most probable cause of this difference may be attributable to the influence of AA10 on parasitaemia development. The CHQ-positive control also showed a similar, albeit higher AUC_0–21 days_ than AA10, trend which effect may also be attributable to the influence of the drug on the percentage parasitaemia. The elongation of the bioavailability of AA in plasma, after oral administration, depends on a number of factors of which the amphiphilic nature of the triterpenoid is a major one [31, 32]. Indeed, AA percentage human intestinal absorption (% HIA) was predicted as 91.23 %, Caco-2 cell permeability as 20.97 nm sec^−1^ and plasma protein distribution as 96.45 %, suggesting well absorptivity, middle permeability and strong binding, respectively [33], which could account for possible AA accumulation in plasma relative to the albumin concentration [34–36]. Indeed, AA bioavailability and accumulation in circulation and tissues has been reported to increase with the duration of AA intake and with possible local and systemic protective effects [37]. This may explain how the parasitaemia was suppressed even when high erythrocytic-phase parasite inoculum (0.6–0.7 mL pRBC suspension), several-fold higher than human infection dose (50–100 μL), was used to establish malaria. An efficient chemoprophylaxis is expected to inhibit establishment of patent malaria. While this did not happen, the later clearance of parasitaemia may indicate that AA has suppressive or clinical prophylaxis and will require certain levels to be reached for efficacy to be achieved.

In the experiment by Yin et al. (2012) the recovery of intact AA from tissues and plasma after dietary intake which reached peak plasma concentrations quickly (0.5 h) after oral intake [37], the rapid metabolic rate of AA in rat liver microsomes and primary hepatocytes (t_1/2_ = 9.493 min) and accompanying low AA bioavailability (16.25 % or 394.2 ng/mL) [38] further indicate that the phytochemical needs time to reach certain lethal levels against malaria. In the current study there was no indication of low bioavailability as the phytochemical managed to retard parasitaemia patency. Oral absorption of AA occurs throughout the small intestines with the highest absorption occurring in the jejunum [38]. Absorption is characterized by two peaks from a single dose inter-spaced by 8 hours, which could be attributable to the enterohepatic circulation [39] and avid binding by albumin [40].

Animals administered with AA (10 mg/kg AA) pre-infection besides suppressing parasitaemia, also preserved food and water intake as well as increased weight gain (Table 1). This may mean that po administration of AA is optimum at 10 mg/kg. Unlike some bitter tasting anti-malarials used for prophylaxis, AA is tasteless having most likely no effect on the brain-gut axis that senses the bitterness, inducing satiety, reduced food and water intake in treated animals [41–43]. Indeed, animals that received CHQ, which is bitter, posted reduced food and water intake as well as weight loss.

Infected animals that were not treated (IC) showed critically low food and water intake as well as negative weight gain. Prolonged reduced food intake results in increased breakdown of stored fat and proteins, production of keto acids with concurrent acidosis and increased oxidative stress (OS). These conditions weaken the animal, promoting parasitaemia, aspects which may have led to the spectacular percentage parasitaemia differences between the AA (10 mg/kg) and the IC. These same effects of hyperparasitaemia were also evident in the animals treated with 30CHQ showing lack of prophylaxis of the drug at this concentration. Furthermore, AA has been reported to have an anti-hyperglycaemic effect through attenuation of glycolytic enzymes and inhibition of glycogen phosphorylase [20]. Asiatic acid was administered in normoglycaemic animals. Consequently, AA inhibition of gluconeogenesis whilst increasing glucose oxidation (upregulation of glycolysis), resulted in energy deficits that could only be satisfied by exogenous sources. Animals administered with AA 10 mg/kg necessarily had to increase food and water intake, which resulted in an increase percentage weight gain, to avert hypoglycaemia. In other words, while the innate immune system combats the infection [44], continued food intake is paramount to alleviate parasitic effects [45] making AA’s ability to increase feeding crucial in malaria [46]. Micronutrient malnutrition has been linked to malaria anaemia pathogenesis [47] and the three (malaria, malnutrition, anaemia) are the common face of childhood disease in many parts of the developing world [48].

To corroborate the reduced food intake was the retardation in RBC mass reduction, as shown by the SMA in thin blood smears (Fig. 1) as well as Hb measurements (Fig. 5), in animals administered with AA 10 mg/kg in comparison to the IC on day 12. There is a contrast between the RBC morphology on day 7 when compared to day 21 for the AA 10 mg/kg administered animals that reflects the slight slump in Hb observed on the earlier time period. Compared to the NIC this change in Hb in AA 10 mg/kg shows that no chemoprophylaxis agent may be 100 % effective all the time [1]. However, the low Hb observed in the IC demonstrates SMA caused by npRBC destruction [49], dyserythropoesis and/or ineffective erythropoiesis [50] and the general cachexia of the inflammatory disease [51] in the absence of effective chemoprophylaxis (Fig. 1c). Driving the hypochromic morphology observed with both IC and CHQ-treated animals (Fig. 1c, d) is a synchronous release of parasite pyrogens such as tumour necrosis factor-α (TNF-α) and interleukin-1β (IL-1β) that are also associated with anaemia, various pathologies and death from malaria [52, 53]. While haemolysis results in reduced RBC mass, the more devastating effect is the release of merozoites which will infect more RBCs, inflammatory chemokines that upregulate leucocytosis, free Hb that rapidly inactivates nitric oxide with concomitant endothelium insults that follow which leads to vasoconstriction and damage to critical organs [54, 55]. Compared to the CHQ-treated and the IC groups, AA administration did avert anaemia showing also that npRBC haemolysis was inhibited and with it, deleterious inflammatory mediators were also suppressed. With malaria-induced anaemia being one of the major contributors to the 43 % anaemia prevalence in children between the age of six and 59 months [56], its inhibition by AA administration from developing in young rats, provides possible leads into preventive malarial disease management.

Elevated leucocytosis, a surrogate marker of inflammation, was observed in the IC and the CHQ-treated animals but not in the NIC or AA (10 mg/kg) administered animals (Fig. 4). In malaria, inflammation is initiated by the release of glycosylphosphatidylinositol (GPI) during or at the end of merogony or RBC death when the pRBCs rapture [51]. The parasite pleiotropic influence-exerting GPI induces high levels of cytokines (TNF-α, IL-1β, IL-6) release from macrophages, causing the pyrexia and cachexia of malaria when it substitutes for the host GPI-based signal transduction in regulating protein kinase C, calcium levels, cell adhesion and nitric oxide (NO) synthesis [57, 58]. Macrophages and polyclonal lymphocyte activation, which was observed as leucocytosis in the IC and CHQ treated animals (Fig. 4) but not in AA-administered animals, may be a reflection of the aberrant GPI hyperactivity and excitation of both pro-inflammatory and anti-inflammatory responses [59] involving the nuclear factor-Kβ (NF-Kβ) [60] in the malaria syndrome. With all this in perspective, it will be safe to infer that the prophylaxis administration of AA (10 mg/kg) did inhibit WBC count increase resulting from reduction in parasite infectivity, merogony abatement and subsequent insufficient GPI release as compared to the IC and CHQ-treated animals.

The founding principles of malaria lie in the successful activation of the immune system, incitement of the inflammatory cascade, abrogation of the haematopoietic function, systemic and endothelial changes with end organ failure, invariably initiated and orchestrated by an obligate intracellular protozoa [61, 62]. Therefore, it stands to reason that chemoprophylaxis approaches of malaria may of necessity focus on the prevention of these abnormalities from developing. While it might be impossible to have 100 % chemoprevention in malaria, infringement on the development of post-infection pathophysiology is crucial in keeping in check overt malaria disease occurrence [63]. Administered before infection or at the onset of the infection, AA10 may be able to avert the development of SM and the accompanying pathophysiology.

## Conclusions

Presented here is data that demonstrate positive AA influence on food and water intake as well as percentage weight gain. Animals administered with AA (10 mg/kg) averted inflammation and severe malaria anaemia development. The anti-parasitic and anti-disease activities of AA in suppressing the parasite while inhibiting infection-induced pathology was evident. Administration of AA (10 mg/kg) showed a suppressive or clinical chemoprophylaxis better than chloroquine at 30 mg/kg, suggesting that AA may be used successfully in the prevention of malaria infection.

## Abbreviations

AA: asciatic acid
ANOVA: analysis of variance
CA: *Centella asiatica*
CHQ: chloroquine
CM: cerebral malaria
DMSO: dimethyl sulfoxide
GPI: glycosylphosphatidylinositol
Hb: haemoglobin
HIA: human intestinal absorption
IC: infected non-treated control
ig: intragastric
ip: intraperitoneal
MA: maslinic acid
OA: oleanolic acid
OS: oxidative stress
po: per-oral
pRBC: parasitized red blood cell
SM: severe malaria
SMA: severe malaria anaemia
WBC: white blood cell

## References

[1] Baker L, Blumberg L, Barnes KI, Hansford F, Duvenage C, Williams HV (2003). Guidelines for the prevention of malaria in South Africa.

[2] Landry PID, Darioli R, Burnier M, Genton B (2006). Do travelers really take their mefloquine prophylaxis? Estimation of adherence by an electronic pill box. J Travel Med.

[3] Senn N, D’Acremont V, Landry P, Genton G (2007). Malaria chemoprophylaxis: what do the travelers choose, and how does pretravel consultation influence their final decision. Am J Trop Med Hyg.

[4] Behrens RH, Taylor RB, Pryce DI, Low AS (1998). Chemoprophylaxis compliance in travelers with malaria. J Travel Med.

[5] Schlagenhauf P, Tschopp A, Johnson R, Nothdurft HD, Beck B, Schwartz E (2003). Tolerability of malaria chemoprophylaxis in nonimmune travellers to sub-Saharan Africa: multicentre, randomised, double blind, four arm study. BMJ.

[6] Ogwang PE, Ogwa JO, Kasasa S, Olila D, Ejobi F, Kabasa D (2012). *Artemisia annua* L. infusion consumed once a week reduces risk of multiple episodes of malaria: a randomised trial in a Ugandan community. Trop J Pharm Res.

[7] Rath K, Taxis K, Walz G, Gleiter CH, Li S, Heide L (2004). Pharmacokinetic study of artemisinin after oral intake of traditional preparation of *Artemisia annua* L.. Am J Trop Med Hyg.

[8] Mbatha B. Treatment of *P. berghei* infected Sprague Dawley rats with oleanolic acid: effects on blood glucose and renal handling. Human Physiology. University of KwaZulu Natal, College of Health Sciences. 2014.

[9] Thaane T. Evaluation of the efficacy of maslinic acid on malaria parasites in *Plasmodium berghei*-infected male Sprague-Dawley rats: effects on blood glucose and renal fluid and electrolyte handling. Human Physiology. University of KwaZulu Natal, College of Health Sciences. 2014.

[10] Lee W, Yang E, Ku SK, Song KS, Bae JS (2013). Anti-inflammatory effects of oleanolic acid on LPS-induced inflammation in vitro and in vivo. Inflammation.

[11] Mkhwanazi BN, Serumula MR, Myburg RB, van-Heerden F, Musabayane CT (2014). Antioxidant effects of maslinic acid in livers, hearts and kidneys of streptozotocin-induced diabetic rats: effects on kidney function. Ren Fail.

[12] Ramachandran V, Saravanan R (2013). Asiatic acid prevents lipid peroxidation and improves antioxidant status in rats with streptozotocin-induced diabetes. J Funct Foods.

[13] Huang SS, Chiu CS, Chen HJ, Hou WC, Sheu MJ, Lin YC (2011). Antinociceptive activities and the mechanisms of anti-inflammation of asiatic acid in mice. Evid Based Complement Alternat Med.

[14] Guo W, Liu W, Hong S, Liu H, Qian C, Shen Y (2012). Mitochondria-dependent apoptosis of con A-activated T lymphocytes induced by Asiatic acid for preventing murine fulminant hepatitis. PLoS ONE.

[15] Patel H, Dhangar K, Sonawane Y, Surana S, Karpoormath R, Thapliyal N et al. In search of selective 11 beta-HSD type 1 inhibitors without nephrotoxicity: an approach to resolve the metabolic syndrome by virtual based screening. Arab J Chem. 2015; in press.

[16] Madlala HP, Masola B, Singh M, Musabayane CT (2012). The effects of *Syzygium aromaticum*-derived oleanolic acid on kidney function of male Sprague-Dawley rats and on kidney and liver cell lines. Ren Fail.

[17] Mapanga RF, Tufts MA, Shode FO, Musabyane CT (2009). Renal effects of plant-derived oleanolic acid in streptozotocin-induced diabetic rats. Ren Fail.

[18] Musabayana CT, Tufts MA, Mapanga RF (2010). Synergistic hypoglycaemic effects between *Syzigium aromaticum*-derived oleanolic acid and insulin in streptozotocin-induced diabetic rats. Soc Endocrinol.

[19] Ramachandran V, Saravanan R (2014). Antidiabetic and antihyperlipidemic activity of Asiatic acid in diabetic rats, role of HMG CoA: in vivo and in silico approaches. Phytomedicine.

[20] Ramachandran V, Saravanan R (2013). Efficacy of asiatic acid, a pentacyclic triterpene on attenuating the key enzymes activities of carbohydrate metabolism in streptozotocin-induced diabetic rats. Phytomedicine.

[21] Gumede B, Folbb P, Ryffela B (2003). Oral artesunate prevents *Plasmodium berghei* Anka infection in mice. Parasitol Int.

[22] Matsuoka H, Yoshida S, Hirai MA, Ishii A (2001). A rodent malaria Plasmodium berghei, is experimentally transmitted to mice by merely probing of infective mosquito, *Anopheles stephensi*. Parasitol Int.

[23] Brown IN, Philips RS (1974). Immunity to *Plasmodium berghei* in rats: passive serum transfer and role of the spleen. Infect Immun.

[24] Changa K-H, Stevenson MM (2004). Malarial anaemia: mechanisms and implications of insufficient erythropoiesis during blood-stage malaria. Int J Parasitol.

[25] Helmi YA, Mohammad NO (2013). *Centella asiatica:* from folk remedy to the medicinal biotechnology-a state revision. Int J Biosci.

[26] Singh S, Gautam A, Sharma A, Batra A (2010). *Centella asiatica (*L*):* a plant with immense medicinal potential but threatened. Int J Pharm Sci Rev Res.

[27] Rest JR (1982). Cerebral malaria in inbred mice, a new model and its pathology. Trans R Soc Trop Med Hyg.

[28] Garnham PC (1965). The structure of early sporogonic stages of *Plasmodium berghei*. Ann Soc Belges Med Trop Parasitol Mycol.

[29] Vincke LH, Bafort F (1968). Results of 2 years of observation of the cyclical transmission of *Plasmodium berghei*. Ann Soc Belges Med Trop Parasitol Mycol.

[30] Weiss ML, Degiusti DL (1964). Modification of a malaria parasite (*Plasmodium berghei*) following passage through tissue culture. Nature.

[31] Agorama B, Woltosza WS, Bolgera MB (2001). Predicting the impact of physiological and biochemical processes on oral drug bioavailability. Adv Drug Deliv Rev.

[32] Martinez MN, Amidon GL (2002). A mechanistic approach to understanding the factors affecting drug absorption: a review of fundamentals. J Clin Pharmacol.

[33] Kartasasmitaa RE, Musofiroh I, Muhtadi A, Ibrahim S (2014). Binding affinity of asiatic acid derivatives design against inducible nitric oxide synthase and ADMET prediction. J Appl Pharm Sci.

[34] Gokara M, Sudhamalla B, Amooru DG, Subramanyam R (2010). Molecular interaction studies of trimethoxy flavone with human serum albumin. PLoS ONE.

[35] Subramanyam R, Gollapudi A, Bonigala P, Chinnaboina M, Amooru DG (2009). Betulinic acid binding to human serum albumin: a study of protein conformation and binding affinity. J Photochemist Photobiol.

[36] Sudhamalla B, Gokara M, Ahalawat N, Amooru DG, Subramanyam R (2010). Molecular dynamics simulation and binding studies of β-sitosterol with human serum albumin and its biological relevance. J Phys Chem B.

[37] Yin M-C, Lin M-C, Mong M-C, Lin C-Y (2012). Bioavailability, distribution, and antioxidative effects of selected triterpenes in mice. J Agric Food Chem.

[38] Yuan Y, Zhang H, Sun F, Sun S, Zhu Z, Chai Y (2015). Biopharmaceutical and pharmacokinetic characterization of asiatic acid in *Centella asiatica* as determined by a sensitive and robust HPLC-MS method. J Ethnopharmacol.

[39] Zheng X-C, Wang S-H (2009). Determination of asiatic acid in beagle dog plasma after oral administration of *Centella asiatica* extract by precolumn derivatization RP-HPLC. J Chromatogr B.

[40] Gokara M, Malavath T, Kalangi SK, Reddana P, Subramanyam R (2014). Unravelling the binding mechanism of asiatic acid with human serum albumin and its biological implications. J Biomolecul Struct Dynam.

[41] Andreozzi P, Sarnelli G, Pesce M, Zito FP, D’alessandro A, Verlezza V (2015). The bitter taste receptor agonist quinine reduces calorie intake and increases the post-prandial release of cholecystokinin in health subjects. J Neurogastroenterol Motil.

[42] Rozengurt E, Sternini C (2007). Taste receptor signaling in the mammalian gut. Curr Opin Pharmacol.

[43] Wu SV, Rozengurt N, Yang M, Young SH, Sinnett-Smith J, Rozengurt E (2002). Expression of bitter taste receptors of the T2R family in the gastrointestinal tract and enteroendocrine STC-1 cells. Proc Natl Acad Sci USA.

[44] Schofield L, Grau GE (2005). Immunological processes in malaria pathogenesis. Nature Rev Immunol.

[45] Etkin NL, Ross PJ, Romanucci-Ross L, Moerman DE, Tancredi LR (1997). Malaria, medicine and meals: a behavioral perspective. The anthropology of medicine.

[46] Green LS, Green L, Danubio M (1997). Modification of antimalarial action of oxidants in traditional cuisines and medicines by nutrients which influence erythrocyte redox status. Adaptation to malaria: the interaction of biology and culture.

[47] Nussenblatt V, Semba RD (2002). Micronutrient malnutrition and the pathogenesis of malarial anemia. Acta Trop..

[48] Kateera F, Ingabire CM, Hakizimana E, Kalinda P, Mens PF, Grobusch MP (2015). Malaria, anaemia and under-nutrition: three frequently co-existing conditions among preschool children in rural Rwanda. Malar J.

[49] Evans KJ, Hansen DS, Van Rooijen N, Buckingham LA, Schofield L (2005). Severe malarial anaemia of low parasite burden in rodent models results from accelerated clearance of uninfected erythrocytes. Blood.

[50] Clark IA, Chaudhri G (1988). Tumour necrosis factor may contribute to the anaemia of malaria by causing dyserythropoiesis and erythrophagocytosis. Br J Haematol.

[51] Schofield L, Hackett F (1993). Signal transduction in host cells by a glycosylphosphatidyllnositol toxin of malaria parasites. J Exp Med.

[52] Kwiatkowski D, Cannon J, Manogue K, Cerami A, Dinarello C, Greenwood B (1989). Tumour necrosis factor production in *falciparum* malaria and its association with schizont rupture. Clin Exp lmmunol.

[53] Kwiatkowski D, Hill A, Sambou I, Twumasi P, Castracane J, Manogue K (1990). TNF concentration in fatal cerebral, non-fatal cerebral, and uncomplicated *Plasmodium falciparum* malaria. Lancet.

[54] Dondorp AM, Pongponratn E, White NJ (2004). Reduced microcirculatory flow in severe *falciparum* malaria: pathophysiology and electron-microscopic pathology. Acta Trop.

[55] Urban BC, Ing R, Stevenson MM (2005). Early interactions between blood-stage plasmodium parasites and the immune system. Curr Top Microbiol Immunol.

[56] Stevens GA, Finucane MM, De-Regil LM, Paciorek CJ, Flaxman SR, Branca F (2013). Global regional, and national trends in hemoglobin concentration and prevalence of total and severe anemia in children and pregnant and non-pregnant women for 1995–2011: a systematic analysis of population representative data. Lancet Glob Health.

[57] Schofield L, Novakovic S, Gerold P, Schwarz RT, McConville MJ, Tachado SD (1996). Glycosylphosphatidylinositol toxin of Plasmodium up-regulates intercellular adhesion molecule-1, vascular cell adhesion molecule-1, and E-selectin expression in vascular endothelial cells and increases leukocyte and parasite cytoadherence via tyrosine kinase-dependent signal transduction. J Immunol.

[58] Tachado SD, Gerold P, Mcconville MJ, Baldwin T, Quilici D, Schwarz RT (1996). Glycosylphosphatidylinositol toxin of *Plasmodium* induces nitric oxide synthase expression in macrophages and vascular endothelial cells by a protein tyrosine kinase-dependent and protein kinase C-dependent signalling pathway. J Immunol.

[59] Krishnegowda G, Hajjar AM, Zhu J, Douglass EJ, Uematsu S, Akira S (2005). Induction of proinflammatory responses in macrophages by the glycosylphosphatidylinositols of *Plasmodium falciparum*: cell signalling receptors, glycosylphosphatidylinositol (GPI) structural requirement, and regulation of GPI activity. J Biol Chem.

[60] Liou H-C (2002). Regulation of the Immune System by NF-kB and IkB. J Biochem Molecul Biol.

[61] Langhorne JF, Ndungu M, Sponaas A, Marsh K (2008). Immunity to malaria: more questions than answers. Nat Immunol.

[62] Miller LH, Baruch DI, Marsh K, Doumbo OK (2002). The pathogenic basis of malaria. Nature.

[63] Miller LH, Ackerman HC, Su X-Z, Wellems TE (2013). Malaria biology and disease pathogenesis: insights for new treatments. Nature Med.

